# The 2023 Impact of Inflammatory Bowel Disease in Canada: The Influence of Sex and Gender on Canadians Living With Inflammatory Bowel Disease

**DOI:** 10.1093/jcag/gwad011

**Published:** 2023-09-05

**Authors:** Laura E Targownik, Natasha Bollegala, Vivian W Huang, Joseph W Windsor, M Ellen Kuenzig, Eric I Benchimol, Gilaad G Kaplan, Sanjay K Murthy, Alain Bitton, Charles N Bernstein, Jennifer L Jones, Kate Lee, Juan-Nicolás Peña-Sánchez, Noelle Rohatinsky, Sara Ghandeharian, Tal Davis, Jake Weinstein, James H B Im, Nazanin Jannati, Rabia Khan, Priscilla Matthews, Tyrel Jones May, Sahar Tabatabavakili, Rohit Jogendran, Elias Hazan, Mira Browne, Saketh Meka, Sonya Vukovic, Manisha Jogendran, Malini Hu, Jessica Amankwah Osei, Grace Y Wang, Tasbeen Akhtar Sheekha, Ghaida Dahlwi, Quinn Goddard, Julia Gorospe, Cyanne Nisbett, Shira Gertsman, James Sousa, Taylor Morganstein, Taylor Stocks, Ann Weber, Cynthia H Seow

**Affiliations:** Division of Gastroenterology and Hepatology, Mount Sinai Hospital, University of Toronto, Toronto, Ontario, Canada; Department of Gastroenterology, Women’s College Hospital, Toronto, Ontario, Canada; Division of Gastroenterology and Hepatology, Mount Sinai Hospital, University of Toronto, Toronto, Ontario, Canada; Department of Medicine, Temerty Faculty of Medicine, University of Toronto, Toronto, Ontario, Canada; Departments of Medicine and Community Health Sciences, University of Calgary, Calgary, Alberta, Canada; SickKids Inflammatory Bowel Disease Centre, Division of Gastroenterology, Hepatology, and Nutrition, The Hospital for Sick Children, Toronto, Ontario, Canada; Child Health Evaluative Sciences, SickKids Research Institute, The Hospital for Sick Children, Toronto, Ontario, Canada; SickKids Inflammatory Bowel Disease Centre, Division of Gastroenterology, Hepatology, and Nutrition, The Hospital for Sick Children, Toronto, Ontario, Canada; Child Health Evaluative Sciences, SickKids Research Institute, The Hospital for Sick Children, Toronto, Ontario, Canada; ICES, Toronto, Ontario, Canada; Department of Paediatrics, Temerty Faculty of Medicine, University of Toronto, Toronto, Ontario, Canada; Institute of Health Policy, Management, and Evaluation, Dalla Lana School of Public Health, University of Toronto, Toronto, Ontario, Canada; Departments of Medicine and Community Health Sciences, University of Calgary, Calgary, Alberta, Canada; Department of Medicine, University of Ottawa, Ottawa, Ontario, Canada; The Ottawa Hospital IBD Centre, Ottawa, Ontario, Canada; Division of Gastroenterology and Hepatology, McGill University Health Centre IBD Centre, McGill University, Montréal, Quebec, Canada; Department of Internal Medicine, Max Rady College of Medicine, Rady Faculty of Health Sciences, University of Manitoba, Winnipeg, Manitoba, Canada; University of Manitoba IBD Clinical and Research Centre, Winnipeg, Manitoba, Canada; Departments of Medicine, Clinical Health, and Epidemiology, Dalhousie University, Halifax, Nova Scotia, Canada; Crohn’s and Colitis Canada, Toronto, Ontario, Canada; Department of Community Health and Epidemiology, University of Saskatchewan, Saskatoon, Saskatchewan, Canada; College of Nursing, University of Saskatchewan, Saskatoon, Saskatchewan, Canada; Crohn’s and Colitis Canada, Toronto, Ontario, Canada; SickKids Inflammatory Bowel Disease Centre, Division of Gastroenterology, Hepatology, and Nutrition, The Hospital for Sick Children, Toronto, Ontario, Canada; Child Health Evaluative Sciences, SickKids Research Institute, The Hospital for Sick Children, Toronto, Ontario, Canada; SickKids Inflammatory Bowel Disease Centre, Division of Gastroenterology, Hepatology, and Nutrition, The Hospital for Sick Children, Toronto, Ontario, Canada; Child Health Evaluative Sciences, SickKids Research Institute, The Hospital for Sick Children, Toronto, Ontario, Canada; SickKids Inflammatory Bowel Disease Centre, Division of Gastroenterology, Hepatology, and Nutrition, The Hospital for Sick Children, Toronto, Ontario, Canada; Child Health Evaluative Sciences, SickKids Research Institute, The Hospital for Sick Children, Toronto, Ontario, Canada; Department of Community Health and Epidemiology, University of Saskatchewan, Saskatoon, Saskatchewan, Canada; SickKids Inflammatory Bowel Disease Centre, Division of Gastroenterology, Hepatology, and Nutrition, The Hospital for Sick Children, Toronto, Ontario, Canada; Child Health Evaluative Sciences, SickKids Research Institute, The Hospital for Sick Children, Toronto, Ontario, Canada; ICES, Toronto, Ontario, Canada; Department of Medicine, McMaster University, Hamilton, Ontario, Canada; Division of Gastroenterology and Hepatology, University Health Network, University of Toronto, Toronto, Ontario, Canada; Department of Gastroenterology, Temerty Faculty of Medicine, University of Toronto, Toronto, Ontario, Canada; Department of Medicine, Temerty Faculty of Medicine, University of Toronto, Toronto, Ontario, Canada; Department of Internal Medicine, Temerty Faculty of Medicine, University of Toronto, Toronto, Ontario, Canada; SickKids Inflammatory Bowel Disease Centre, Division of Gastroenterology, Hepatology, and Nutrition, The Hospital for Sick Children, Toronto, Ontario, Canada; Child Health Evaluative Sciences, SickKids Research Institute, The Hospital for Sick Children, Toronto, Ontario, Canada; Department of Internal Medicine, Temerty Faculty of Medicine, University of Toronto, Toronto, Ontario, Canada; Department of Internal Medicine, Temerty Faculty of Medicine, University of Toronto, Toronto, Ontario, Canada; Department of Medicine, Queen’s University, Kingston, Ontario, Canada; Department of Gastroenterology, Temerty Faculty of Medicine, University of Toronto, Toronto, Ontario, Canada; Department of Community Health and Epidemiology, University of Saskatchewan, Saskatoon, Saskatchewan, Canada; Department of Medicine, Temerty Faculty of Medicine, University of Toronto, Toronto, Ontario, Canada; Department of Community Health and Epidemiology, University of Saskatchewan, Saskatoon, Saskatchewan, Canada; Division of Gastroenterology, Hepatology, and Nutrition, The Hospital for Sick Children, University of Toronto, Toronto, Canada; Department of Pediatrics, Faculty of Medicine, King Abdulaziz University, Jeddah, Saudi Arabia; Departments of Medicine and Community Health Sciences, University of Calgary, Calgary, Alberta, Canada; Departments of Medicine and Community Health Sciences, University of Calgary, Calgary, Alberta, Canada; Faculty of Law, University of Victoria, Victoria, British Colombia, Canada; School of Criminology, Simon Fraser University, Burnaby, British Colombia, Canada; Michael G. DeGroote School of Medicine, McMaster University, Hamilton, Ontario, Canada; Crohn’s and Colitis Canada, Toronto, Ontario, Canada; Faculty of Medicine and Health Sciences, McGill University, Montreal, Quebec, Canada; Crohn’s and Colitis Canada, Toronto, Ontario, Canada; Department of Obstetrics and Gynecology, Memorial University of Newfoundland, St. John’s, Newfoundland, Canada; Departments of Medicine and Community Health Sciences, University of Calgary, Calgary, Alberta, Canada; Department of Neuroscience, McGill University, Montreal, Quebec, Canada

**Keywords:** Crohn’s disease, Healthcare access, Healthcare utilization, Pregnancy, Ulcerative colitis

## Abstract

Sex (the physical and physiologic effects resulting from having specific combinations of sex chromosomes) and gender (sex-associated behaviours, expectations, identities, and roles) significantly affect the course of inflammatory bowel disease (IBD) and the experience of living with IBD. Sex-influenced physiologic states, like puberty, the menstrual cycle, pregnancy, and andropause/menopause may also impact and be impacted by IBD.

While neither Crohn’s disease nor ulcerative colitis is commonly considered sex-determined illnesses, the relative incidence of Crohn’s disease and ulcerative colitis between males and females varies over the life cycle. In terms of gender, women tend to use healthcare resources at slightly higher rates than men and are more likely to have fragmented care. Women are more commonly prescribed opioid medications and are less likely than men to undergo colectomy. Women tend to report lower quality of life and have higher indirect costs due to higher rates of disability. Women are also more likely to take on caregiver roles for children with IBD. Women with IBD are more commonly burdened with adverse mental health concerns and having poor mental health has a more profound impact on women than men.

Pregnant people with active IBD have higher rates of adverse outcomes in pregnancy, made worse in regions with poor access to IBD specialist care. The majority of individuals with IBD in Canada do not have access to a pregnancy-in-IBD specialist; access to this type of care has been shown to allay fears and increase knowledge among pregnant people with IBD.

**Key Points**

1. There is a clear impact of sex and gender on IBD, and the disease experience. For example, females who present with IBD symptoms can have those symptoms misattributed to ascribed sex/gender issues (e.g., menstrual cramping) and men are less likely to seek healthcare.
2. Research on sex and gender in IBD is lacking. Specifically, how conformity to masculine, feminine, or nongender-normative roles impacts IBD diagnosis and healthcare-seeking behaviour.
3. Females with IBD are about 50% more likely than males with IBD to report anxiety or depression.
4. Pregnancy is associated with greater risks in people with IBD. All individuals with IBD should have access to IBD specialists who focus on preconception counselling and management of IBD during pregnancy, and who collaborate with maternal-fetal medicine specialists and obstetricians who are experienced in the obstetrical management of individuals with IBD.
5. It is important to deeply consider your disease before, during, and after pregnancy and engage your gastroenterologist as a member of your pregnancy team.

## INTRODUCTION

Sex refers to the biological and physiological differences that occur between individuals as a result of the direct or indirect effects of their sex chromosomes. Gender refers to the identity of the individual and how they express themselves in the world.

In this chapter, we summarize the impacts of sex and gender on the disease course and experience of persons living with inflammatory bowel disease (IBD). We provide a framework to understand the work that remains to be done in this area and the role Crohn’s and Colitis Canada and its membership can play in promoting research and mitigating gender-based inequities. We use the terms male/female when describing sex-related influences and men/women when describing gender-related influences. When a study does not explicitly differentiate between sex and gender or when it uses the terms interchangeably, we use sex (male/female) when the outcomes are related to the aetiology, physiology, or objective measures of disease and gender (men/women) when the outcome is primarily behavioural or related to the lived experience of having IBD. We recognize that there are more than two genders; currently, however, research extending to non-gender-conforming individuals with IBD is lacking.

## WHAT IS THE IMPACT OF SEX AND GENDER ON THE EPIDEMIOLOGY OF IBD?

Unlike in many autoimmune diseases, sex does not have a strong impact on the overall risk of developing IBD. In Canada, the ratio of Crohn’s disease between females and males is approximately 1.3 to 1 and ulcerative colitis rates are roughly equal between the sexes (1,2). However, this ratio varies over the life cycle; males are more likely than females to be diagnosed with Crohn’s disease prior to adolescence and females are more commonly diagnosed in adulthood (3). Rates of ulcerative colitis among males and females are similar until late adulthood when a male predominance in diagnosis begins to emerge (3). Although these differences are relatively small, this observation may provide important insights into how age-related variations in sex hormone levels or other sex-related differences may impact the development of IBD. Data from animal studies have suggested that sex hormones, particularly estrogens, can promote certain inflammatory pathways in the intestine. In humans, it is unclear the extent to which exposure to sex hormones, whether internally produced by reproductive organs, or in the form of medications or supplements, affects the course of IBD or impacts inflammatory pathways in a clinically significant way (4–6).

Sex hormones can affect gastrointestinal motility and pain sensitivity. It has been observed that worsening of gastrointestinal symptoms in the premenstrual or menstrual cycle can occur in people with IBD and this may affect the clinical assessment of IBD activity (7–12).

Oral contraceptive use has been associated with an increased risk of being diagnosed with IBD (13), though it is possible that the higher risk of IBD is due to other factors or behaviours that are themselves associated with oral contraceptive use (e.g., higher socioeconomic status or more frequent physician visits and diagnostic testing).

Similarly, gender-related factors may also influence the epidemiology of IBD through the impact that differences in the likelihood of obtaining evaluations and testing have on the timeliness of diagnosis. While there is not yet any definitive evidence on the impact of gender on diagnostic delay (14,15), women are more likely to present with abdominal pain that is presumed to be due to functional disease or psychosomatic rather than due to organic illness (16,17). Conversely, behavioural expectations around stoicism and toughness in men may prevent them from seeking healthcare for gastrointestinal symptoms that may prove to be IBD, though this has not been directly assessed (18). Further research is required to understand how gender and more specifically, how conformity to masculine or feminine gender roles impacts IBD diagnosis and healthcare-seeking behaviour. There is no clear or consistent evidence suggesting that sex or gender influences the incidence or severity of any IBD-specific complication, or is associated with any particular disease phenotype or characteristic (19).

## THE IMPACT OF IBD IN LESBIAN, GAY, BISEXUAL, TRANSGENDER, QUEER, TWO SPIRITED PERSONS (LGBTQ2S+)

When discussing the LGBTQ2S+ community, it is important to differentiate between gender identity, sexual orientation, attraction, and sexual behaviour. As an example, a person assigned male at birth (sex), may see himself as a heterosexual man (sexual orientation and gender identity), be attracted primarily to women (heterosexual attraction), but participate in anal receptive intercourse (sexual behaviour). Each gender identity, sexual orientation, attraction, and behaviour could conceivably have influences on all facets of IBD, from the risk of developing IBD, to how IBD care is accessed, to one’s lived experience in managing and mitigating the effects of IBD.

People in the LGBTQ2S+ populations are more likely to be socioeconomically disadvantaged compared to the general population, which can affect their ability to access healthcare. These disadvantages are further compounded by negative experiences with the healthcare system and fears of further stigmatization, which often leads to delayed diagnosis and higher morbidity in LGBTQ2S+ persons than in the general population. These effects are even more pronounced among people with nonconforming gender identities and may be further aggravated by the intersectional effects of race, ethnicity, and immigration status (20–22).

A recent study using secondarily collected data suggested that men who were reported as practicing high-risk sexual behaviours with other men were more likely to develop Crohn’s disease and ulcerative colitis than were men who practiced high-risk sexual behaviour with women (Crohn’s disease, odds ratio [OR]: 1.64; 95% CI: 1.29, 2.09; ulcerative colitis, OR: 2.45; 95% CI: 2.35, 3.34) (23). This study, however, was criticized for conflating sexual behaviour with sexual identity and was commonly misreported in the lay media as a link between being gay and developing IBD (24). A more recent analysis using data from the United States National Health Interview Survey showed no association in men or in women between sexual orientation and IBD prevalence (Newman K. and Targownik L.E., unpublished data).

A qualitative study that gathered the experiences of gay and lesbian individuals with IBD identified a set of unique concerns of this population related to the stigmatization of gay and lesbian identities, as well as the impact of sexual behaviours associated with same-sex attraction on IBD. Specifically, men who have sex with men commented on the impact of living with a stoma or having perianal disease on their ability to participate in anal receptive intercourse, as well as the impact on body image and dealing with misconceptions about the role their sexual behaviours had on their developing IBD (25).

The epidemiology and natural history of IBD in transgender and gender nonconforming (TG/NC) populations remain unreported. Studies on the lived experience of TG/NC persons with IBD are ongoing, and guidelines for the care of TG/NC adolescents have been proposed (26). IBD-related concerns that may be specific to the TG/NC community include the impact of gender-affirming hormonal therapy on IBD, the effect that perianal disease may have on the ability to undergo gender-affirming surgeries, and on the ability of their IBD care provider to provide culturally competent care in a welcoming and nonstigmatizing environment (27).

## THE IMPACT OF SEX AND GENDER ON HEALTHCARE UTILIZATION

Dittrich et al. assessed individuals with Crohn’s disease in Edmonton, Alberta showing both the annual likelihood of undergoing surgery among males and females was similar (approximately 5.8% per year in 1996; approximately 1.3% per year in 2013) and the change in the incidence of surgery in males (8.5% per year decline; 95% CI: 6.9%, 10.0%) and females (8.4% per year decline; 95% CI: 7/0%, 9.8%) from 1996 to 2013 were similar (28). In a study using Ontario health administrative databases, females with IBD were less likely to receive high exposure to cumulative ionizing radiation (OR: 0.91; 95% CI: 0.87, 0.95); the authors concluded that this may reflect hesitancy to be exposed to radiation in those of childbearing age, rather than discrepant access to care (29).

Analyses performed on population-based data from Manitoba did not demonstrate any differences between males and females in the likelihood of being prescribed a biologic agent or in persistence with biologic therapy once prescribed (30,31). However, in a different dataset, females were more likely than males to discontinue anti-TNF therapy due to subjective drug-related side effects (adjusted hazard ratio [aHR]: 4.05; 95% CI: 2.36, 6.98) (32); similar findings were also seen in a Swedish registry of vedolizumab users (33). There have been few studies which have sought to evaluate gender-based differences in treatment response to biologic therapies *a priori*. Secondary analyses of individual studies have not generally shown consistent impacts of gender on treatment response (34,35). Two studies have suggested that men receiving golimumab or vedolizumab may have lower rates of response to induction therapy, but that this effect dissipates by the end of the maintenance phase (36). The reasons for this difference in speed of response are not well characterized.

Targownik et al. used a population-based IBD registry in Manitoba to assess direct costs attributable to caring for individuals with IBD between 2005 and 2015, as well as the inpatient, outpatient, and surgical visits associated with IBD (37). In a regression analysis, men were admitted to hospital at 80% of the rate of women and sought outpatient care for IBD 7% less frequently than women. However, men were admitted to hospitals for surgical care 18% more often than women (37). Direct costs for males were $762 more per year than for women with IBD (95% CI: $440, $1,085). It is unclear whether this cost is driven by higher surgical costs, more frequent use of diagnostic testing, or greater use of costly biologic medications. The average Canadian male outweighs the average Canadian female by 15 kg (38), resulting in higher costs for drugs with weight-based dosing (e.g., biologics). While there have been multiple other population-based studies detailing the direct costs of care associated with IBD in North America and Europe, none specifically describe gender-based differences in healthcare spending (See Kuenzig et al., in this volume).

Shafer et al. reported that although higher levels of disability were associated with greater income loss, there was no analysis evaluating whether the impact of disability on income is different between women and men (39). The impact of disability on income is important to better characterize as multiple European studies have suggested that women reported greater disability associated with IBD than men. In Sweden, women with IBD were shown to earn less than their age- and sex-matched siblings in the five years following diagnosis (income loss of €9,778 vs. €6,569) (40). In the same dataset, Swedish women reported a greater number of missed days of work than men, especially in the peri-diagnosis time period (41). However, women did not appear to have higher rates of income loss in the Danish cohort (42). Importantly, women who served as primary caregivers for dependents with IBD were more likely to perceive high levels of caregiver burden, which predicted higher rates of absenteeism and presenteeism (See Kuenzig et al., in this volume) (43).

## THE IMPACT OF SEX AND GENDER ON MENTAL HEALTH AND QUALITY OF LIFE IN IBD

Mental health concerns are consistently identified by individuals with IBD as a priority. Health-related quality of life refers to the individual’s subjective sense of well-being and is driven by the burden of disease-associated symptoms, a person’s mental health, and the impact that physical and mental health have on a person’s ability to socially and economically engage with their families and communities. International consensus panels and afflicted individuals agree that improving the overall quality of life is a critical treatment goal in IBD (44), and that care providers should be striving not only to improve symptoms and inflammation but working with individuals with IBD to optimize their overall sense of well-being.

There are limited data to support whether there are differences in the overall burden of symptoms associated with IBD, controlling for the underlying severity of IBD (45). Women do tend to report more fatigue, which is a major driver of decreased quality of life in IBD and is associated with poorer mental health and decreased workplace engagement (46). Different factors may be driving this, including the higher prevalence of iron deficiency among females, the impacts of hormones and hormonal fluctuations, or a higher prevalence of concomitant disorders of gut–brain interaction (47).

Many women with IBD also report changes in symptoms ascribed to IBD that occur at or before the time of menses; however, there have not been any studies that have associated this change in symptoms with the level of inflammatory activity or directly to fluctuations in hormone levels (9,12). Perimenstrual gastrointestinal symptoms are frequently reported by people without IBD as well, and data about whether perimenstrual gastrointestinal symptoms are more severe among individuals with IBD are lacking (8). With regards to the impact of concomitant disorders of gut–brain interaction (DGBI), Bryant et al. reported a higher rate of anxiety (78% vs. 22%) and depression (89% vs. 11%, both *P* < 0.001) among women with DGBIs along with IBD and female sex as an independent predictor of having a DGBI in IBD (OR: 2.17; 95% CI: 1.02, 4.55) (48).

Women with IBD are about 50% more likely than men with IBD to report anxiety or depression (49,50). In 2019, Lewis et al. used the University of Manitoba Research Registry to identify people with currently active or previously active anxiety and depression (51). Among 242 persons with IBD, 40% and 30% reported a history of depression and anxiety, respectively, of whom 11% and 17% reported their depression or anxiety being currently active. Men were more likely than women to state that they had not reported having depression to their physicians. These findings highlight the importance of a systematic approach to addressing mental health concerns among persons with IBD and not relying on individuals to self-report or relying on gender-influenced stereotypes to determine which individuals may have a mental illness. An analysis of the population-based University of Manitoba IBD Epidemiologic Registry found that women with anxiety/depression had 1.23 additional ambulatory care visits when compared with men; however, they did not explore whether excess healthcare utilization in persons with anxiety/depression was influenced by gender (52).

Narula et al. explored the relationship between elevated hospital anxiety and depression scores (HADS), a commonly used questionnaire to screen for anxiety and depression, in the subsequent course of IBD (53). They determined that having an elevated HADS, particularly for anxiety, was associated with higher rates of a more severe IBD course. There was no difference based on gender in HADS and sex/gender did not influence the likelihood of having a more severe disease course. However, this study did not specifically assess whether sex/gender differences in individuals with adverse mental health impacted their likelihood of having adverse IBD outcomes.

The mental health of persons with IBD is addressed in detail in Graff et al., in this volume.

## PREGNANCY AND IBD

The majority of people with IBD are diagnosed during early adulthood and adolescence, meaning that they will be living with IBD during their prime reproductive years. People with IBD have concerns about the impact of the IBD diagnosis, IBD phenotype, and IBD disease activity on fertility and pregnancy outcomes, as well as delivery methods. They report concerns and a lack of knowledge regarding the uncertainty of the use of IBD medications during preconception and pregnancy (54,55). Therefore, it is recommended that individuals with IBD who are contemplating pregnancy or who are pregnant receive individualized education and counselling regarding the management of their IBD during this time. They should have access to clinicians (gastroenterologists and obstetricians) who are experienced and knowledgeable in the management of IBD during pregnancy.

Among the IBD population, there is a high rate of observed voluntary childlessness, and up to 17% of surveyed females report choosing to remain childless (56). Factors associated with voluntary childlessness were poorer reproductive knowledge (as measured by the Crohn’s Colitis Pregnancy Knowledge score), older age, unemployment, being single, and not seeking medical advice (56). Lack of knowledge regarding reproductive health issues in IBD results in fears and uncertainty regarding having a diagnosis of IBD, taking IBD therapies, and pregnancy outcomes (57). About one-third of females considering pregnancy reported stopping their medications without discussing them with their physicians (58); this seems to be a result of concerns about safety and uncertainty about the medications with pregnancy (59). Dedicated clinical counselling and education can increase pregnancy-specific knowledge (58–60). Preconception counselling has demonstrated benefits, including reducing IBD relapse in pregnancy by increasing adherence to medications and smoking cessation (61). A subset of individuals with IBD who have had surgery may be at increased risk of miscarriage, requiring assisted reproductive therapies, caesarean section delivery, having a low birth weight infant, and possible infertility (62). Persons with IBD, especially Crohn’s disease, are less likely to become pregnant or carry a pregnancy to term when compared to age-matched controls, particularly around the time of diagnosis. Having a history of a pouch procedure may also reduce birth rates and pregnancies in persons with ulcerative colitis by around 50% (63,64).

Females with IBD, especially those with active or complex disease, are at increased risk for adverse pregnancy outcomes, including pregnancy loss, preterm birth, small for gestational age (SGA) infants, low birth weight infants, and caesarean delivery, as well as complications such as venous thrombosis. Predictors of disease activity in pregnancy include a history of disease activity in a prior pregnancy (OR: 4.21; 95% CI: 1.10, 16.58) (65) and active disease at conception (aOR: 7.66; 95% CI: 3.77, 15.54) (66). Individuals with these risk factors may benefit from specialized counselling and optimal management strategies in preconception and pregnancy time. Specifically, pregnant individuals who have moderate or severe IBD and are at increased risk for adverse pregnancy outcomes may benefit from this model of care to attempt to reduce their increased risk for adverse outcomes to be comparable to those with milder IBD or the general population (67). Females with IBD were also at increased risk of developing postpartum depression and anxiety (68). Multidisciplinary shared care with gastroenterologists and obstetricians/maternal-fetal medicine specialists who specialize in the care of pregnant people with IBD may benefit this population.

Many individuals with IBD do not have consistent access to IBD-specific preconception and pregnancy care and are not receiving frequent counselling about IBD and reproductive health issues (58). A cross-sectional survey in the United Kingdom reported variation in the prenatal services provided by IBD units, where only 14% of IBD prenatal care was provided by a gastroenterologist with expertise in pregnancy and only 14% of units offered combined clinics with obstetricians and gastroenterologists (69). In an Ontario population-based cohort study, the highest rates of adverse outcomes (preterm delivery, aOR: 2.78; 95% CI: 1.03, 7.46; SGA infants, aOR: 5.66; 95% CI: 1.67, 19.14; Caesarean section aOR: 2.48; 95% CI: 1.11, 5.55) were observed in individuals in the most northern rural areas, with no differences observed in central urban health units (70).

Recent international guidelines such as those from the British Society of Gastroenterology and British Maternal & Fetal Medicine Society recommend collaborative care between obstetrics and IBD units or coordinated communication to provide preconception counselling to all females with IBD and counselling to pregnant people on the safety of IBD medication during pregnancy and breastfeeding, the optimal mode of delivery and the management of biologics and childhood vaccines(71).

All individuals with IBD should have access to IBD specialists who focus on preconception counselling and management of IBD during pregnancy and who collaborate with maternal-fetal medicine specialists and obstetricians who are experienced in the obstetrical management of individuals with IBD. As there may not be a sufficient number of IBD specialists with this expertize, Canada should consider cross-provincial resources where consultants may offer support in other provinces.

## CONCLUSION

Despite the centrality of sex and gender in our day-to-day experience, there has been relatively little research that is focused on the specific ways that sex or gender impacts IBD. There is an increasing recognition of the importance of sex- and gender-related factors on health and disease and many funding agencies (including the Canadian Institutes of Health Research and Crohn’s and Colitis Canada) compel research applicants to describe how sex- and gender-related determinants will be included as part of the research design. However, assessing sex- and gender-related influences are often not the primary question being studied. In addition, the interactions that sex- and gender-related factors have on known associations (e.g., adverse mental health or worse IBD outcomes) are rarely explored.

Due to the high prevalence of IBD in Canada, well-organized datasets and a highly engaged patient/physician/researcher community, Canadians are well-positioned to conduct, coordinate, and disseminate higher-quality research in this area.

## KNOWLEDGE GAPS AND FUTURE RESEARCH DIRECTIONS

1. There is limited knowledge as to the experience and healthcare needs of transgender and gender-diverse individuals living with IBD.
2. Further data confirming the value of comprehensive care clinics targeting females with high risk IBD who are or are seeking to become pregnant are required.
3. There are limited data on the impact of cyclical and lifetime hormonal changes on the etiology of IBD, symptom burden, and intestinal inflammation, which also needs to include cyclic hormones around menstruation and hormone replacement therapy.

## PATIENT AND CAREGIVER PARTNER PERSPECTIVE

Due to a paucity of evidence presented in this chapter, patient partners consider it critical to continue promoting studies that understand how disease course, healthcare utilization, and experiences are affected by sex and gender, as well as research initiatives that identify strategies that could address existing gender inequities. For example, patient partners highlighted the need for evidence expanding our understanding of mental health differences by gender, experiences of transgender and gender-diverse individuals living with IBD, how masculine or feminine gender roles impact IBD diagnosis and healthcare-seeking behaviours, why certain gender groups face barriers to access to IBD care, and sex and sexual health among individuals with IBD, among other topics. In addition, patient partners appreciated reading what is known about IBD and pregnancy but felt more research was needed. Understanding how this condition could affect their pregnancy and vice versa, as well as learning about the use of medications for IBD during pregnancy is fundamental for persons living with IBD who are considering parenthood. Patient partners noted conversations surrounding gender issues, sexual health, and pregnancy planning rarely happen between persons living with IBD and gastroenterology care providers. The IBD community should advocate for access to preconception and prenatal counselling/education for individuals living with IBD and to the care of gastroenterologists and obstetricians with expertize in IBD during and after pregnancy, including cross-provincial models of healthcare that allow for sharing of expertize to more rural and remote areas.

## POLICY IMPLICATIONS AND KEY ADVOCACY OUTCOMES

1. Advocacy should aim to support research specifically addressing sex- and gender-related factors on the course of IBD, on the lived experience of IBD, and how to best minimize disparities based on sex and/or gender—including nonbinary and other genders. A strong knowledge translation focus on this research will inform clinicians and may positively affect the quality of care they provide.
2. Research should aim to discover any correlations between sex (as reported in administrative healthcare databases) and gender (as identified by the individual).
3. Advocacy should continue to seek multidisciplinary care for those with IBD who are seeking to become pregnant and support the mental and sexual health of Canadians living with IBD. This advocacy should include resources to match these individuals with OBGYN specialists who are knowledgeable in IBD care (e.g., databases, programs, and inter-provincial collaboration).
4. Crohn’s and Colitis Canada should advocate for a safe space in-hospitals and clinics and produce educational material (e.g., brochures, posters) for practitioners with resources/tips for creating a safe-space atmosphere, including information on differentiating common IBD symptoms from sex-specific symptoms (e.g., menstrual pains, endometriosis).
5. Crohn’s and Colitis Canada should expand advocacy around washroom access to include advocacy for washroom access for transgendered individuals and those who do not identify as male or female.

## SUPPLEMENT SPONSORSHIP

This article appears as part of the supplement “The Impact of Inflammatory Bowel Disease in Canada in 2023”, sponsored by Crohn’s and Colitis Canada, and supported by Canadian Institutes of Health Research Project Scheme Operating Grant (Reference number PJT-162393).

## Data Availability

No new data were generated or analyzed in support of this review.
