# Correction to: The 2023 Impact of Inflammatory Bowel Disease in Canada: The Influence of Sex and Gender on Canadians Living With Inflammatory Bowel Disease

**DOI:** 10.1093/jcag/gwae012

**Published:** 2024-03-08

This is a correction to: Laura E Targownik, *et al*, The 2023 Impact of Inflammatory Bowel Disease in Canada: The Influence of Sex and Gender on Canadians Living With Inflammatory Bowel Disease, *Journal of the Canadian Association of Gastroenterology*, Volume 6, Issue Supplement_2, September 2023, Pages S55–S63, https://doi.org/10.1093/jcag/gwad011

In the originally published version of this manuscript, the author Vivian W. Huang was incorrectly listed as Vivian H. Huang.

This error has been corrected online.

